# Infective Endocarditis Caused by Streptococcus sinensis: A Rare but Fatal Infection

**DOI:** 10.7759/cureus.70444

**Published:** 2024-09-29

**Authors:** Nicholas Tabone Adami, James Farrugia, Angel Galdes

**Affiliations:** 1 Internal Medicine, Mater Dei Hospital, L-Imsida, MLT; 2 Infectious Disease, Mater Dei Hospital, L-Imsida, MLT; 3 Microbiology, Mater Dei Hospital, L-Imsida, MLT

**Keywords:** infective endocarditis, maldi tof, maltese islands, novel strains, strep viridian

## Abstract

We present a case concerning a 65-year-old, previously healthy gentleman who was found to have infective endocarditis after presenting with a four-month history of constitutional symptoms. Blood tests taken in the community showed elevated inflammatory markers and a deterioration in renal function, which prompted his referral for inpatient investigations. Blood cultures and a transesophageal echocardiogram subsequently confirmed native valve endocarditis affecting both the aortic and mitral valves. Infective endocarditis is common in an infectious disease physician’s daily practice, but this particular case is notable since the causative agent, *Streptococcus sinensis*, is an extremely rare viridans group *Streptococcus*, which has been reported only sporadically in other European countries. The patient was started on appropriate anti-microbial treatment and was scheduled for surgery to replace both affected valves. He developed signs and symptoms of congestive heart failure after 10 days of anti-microbial treatment and underwent dual valve replacement surgery. Despite optimal anti-microbial therapy and multiorgan support in a cardiac intensive care unit, the patient passed away two weeks after surgery as a result of ventilator-associated pneumonia.

## Introduction

Infective endocarditis is a rare infection involving the endocardial surface of the heart, typically affecting one or more of the cardiac valves. The condition often presents with non-specific symptoms, including lethargy, night sweats, and unexplained fever. Clinical examination findings suspicious for infective endocarditis include a new murmur, the presence of nail-bed splinter hemorrhages, Osler nodes and Janeway lesions in the peripheries, and Roth spots on fundoscopy.

The condition is also associated with a multitude of complications, including congestive heart failure and the formation of immune complexes and systemic emboli. Duke criteria may be applied when a high clinical suspicion of infective endocarditis arises [1]. The criteria take into consideration the presence of positive blood cultures with typical microorganisms and evidence of endocardial involvement on echocardiography as the major criteria. The minor criteria include the presence of a fever, vascular or immunologic phenomena, and a history of predisposing heart disease or intravenous drug abuse.

The microorganisms that most commonly cause endocarditis include *Staphylococci*, *Enterococci*, and *Streptococci*. Less commonly, organisms such as the HACEK organisms (*Haemophilus*, *Actinobacillus*, *Cardiobacterium*, *Eikenella*, and *Kingella*) can be identified as the cause of infective endocarditis. Risk factors for developing community-acquired infective endocarditis include immunosuppression, intravenous drug use, poor dentition, and rheumatic heart disease. Viridans group *Streptococci* are responsible for about 20% of cases of community-acquired infective endocarditis [2].

This case is notable because it describes infective endocarditis caused by *Streptococcus (S.) sinensis*, a bacterium first isolated in China in 2000 [3]. It is an extremely rare, emerging agent of infective endocarditis in Europe, with only sporadic cases reported in Italy, France, and the Netherlands.

Management of native valve infective endocarditis includes multidisciplinary care by infectious disease specialists, cardiologists, and cardiothoracic surgeons. Prompt diagnosis and initiating appropriate antimicrobial therapy are essential in reducing complications. Antibiotic therapy is usually required for four to six weeks. In patients with complications such as severe valve dysfunction or poor response to antibiotics, surgical intervention may be necessary to replace the affected valve.

## Case presentation

A 65-year-old, Caucasian, southern Mediterranean male presented to casualty with a four-month history of worsening lethargy, weight loss, and poor appetite. Blood investigations taken in the community also showed a sudden deterioration in renal function. The patient’s history was notable for a dental scaling procedure performed several weeks before the onset of symptoms. He also had a history of colon cancer that was treated by surgical resection, with no evidence of recurrence. He did not have any recent travel history of note.

Physical examination revealed a cachectic and pale patient with a low-grade fever of 37.6 °C. He was normotensive and maintained adequate saturations on room air. Multiple splinter hemorrhages were noted on inspection of the hands. Examination of the cardiovascular system was notable for a diastolic murmur in the aortic area and a pan-systolic murmur in the mitral area radiating to the axilla. His chest was clear on examination and jugular venous pressure was not raised. Bilateral pitting ankle edema was noted. The patient’s physical and biochemical parameters were monitored daily along with a focused physical examination to assess for signs of heart failure.

**Figure 1:**
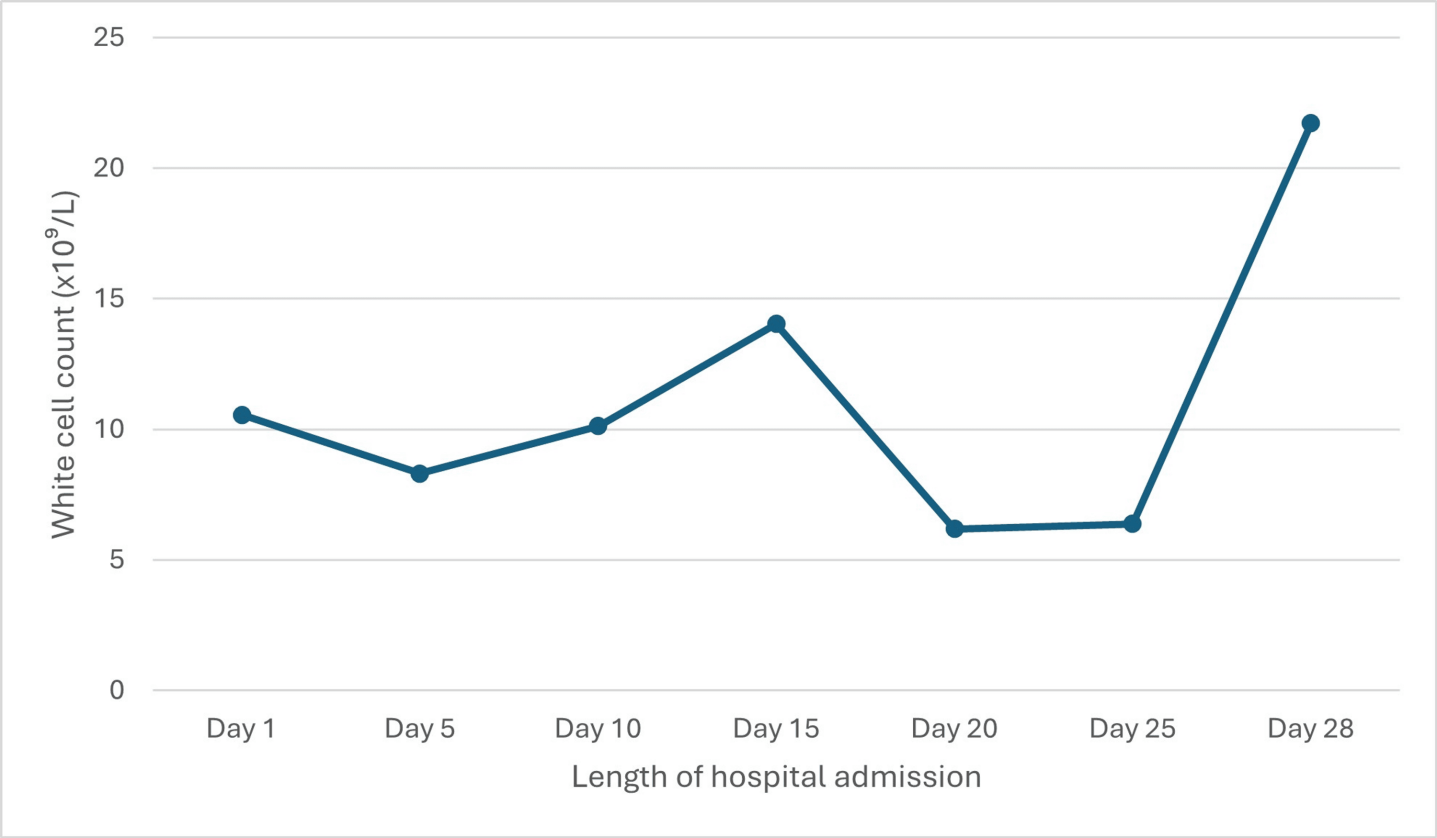
Graph indicating the fluctuation in white cell count during the course of hospital admission

**Figure 2:**
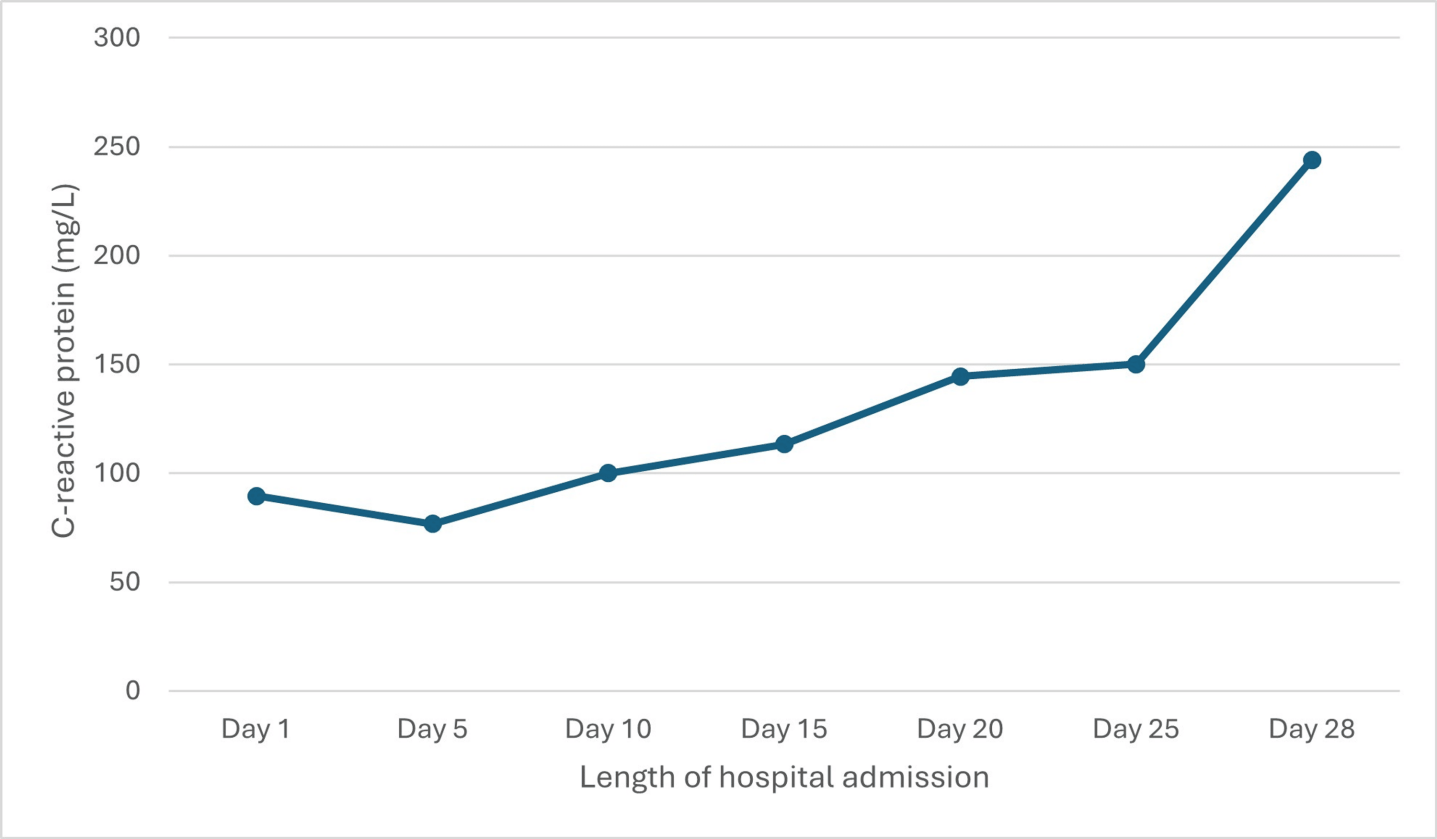
Graph showing the gradual rise in C-reactive protein levels during the course of hospital admission

Following 10 days of intravenous antibiotic therapy, the patient developed new oxygen requirements and dyspnea on minimal exertion. He was started on intravenous bumetanide to good effect. However, because the patient developed signs of progressive heart failure, valve replacement surgery was expedited. Following surgical intervention, the patient was managed in a cardiac intensive care unit, requiring inotropic support, intubation with ventilatory support, and intermittent hemodialysis. Surgery was complicated by an intra-mediastinal bleed, which required re-operation several hours after the procedure. Despite an initial improvement in cardiac function with a gradual reduction in the requirement for inotropic support, a rise in inflammatory markers was noted, with a white cell count of 15.4 x10⁹/L and a C-reactive protein of 244 mg/L, as shown in Figure 1 and Figure 2, respectively. Repeat chest X-ray was in keeping with ventilator-associated pneumonia.

The antibiotic spectrum was broadened to include hospital-acquired organisms while blood cultures and sputum cultures were taken. High-dose co-trimoxazole was added to treatment once *Strenotrophomonas maltophilia* was cultured from sputum. Unfortunately, the patient passed away two weeks after surgery.

Preliminary blood investigations revealed an acute kidney injury with a serum creatinine of 207 µmol/L and a serum urea of 14.8 mmol/L. Inflammatory markers were raised, with a C-reactive protein of 89.6 mg/L. Given that this patient was febrile and had a new heart murmur, blood cultures were taken and were found to be positive for *Streptococcus sinensis* (penicillin minimum inhibitory concentration (MIC) 0.064).

Given the high suspicion of infective endocarditis, a trans-thoracic echocardiography (TTE) was performed, which showed a mass on the aortic valve associated with severe aortic regurgitation and moderate aortic stenosis. The ejection fraction was found to be preserved at 61%. In addition, severe mitral regurgitation was noted. The left atrium was found to be severely dilated with dimensions of 51 ml/m² and a mildly dilated left ventricle, with an end diastolic volume of 81 ml/m².

A trans-esophageal echocardiogram (TOE) was then performed because it visualizes the aortic root and posterior structures, such as the mitral valve, with superior resolution when compared to TTE. The TOE confirmed the presence of a large vegetation on the right coronary cusp causing moderate aortic stenosis, and further vegetation on the anterior mitral valve leaflet causing severe mitral regurgitation. The aortic root was found to be of normal size, and there was no evidence of aortic root abscess on TOE.

In view of the worsening renal function with the reducing urine output, a urinalysis was taken, which revealed an erythrocyte count of 250 uL and a protein of 500 mg/dl. The urinary albumin-to-creatinine ratio confirmed nephrotic range proteinuria. These findings were in keeping with glomerulonephritis, most likely secondary to renal immune complex deposition.

Microbiology investigations

The patient’s blood was injected into aerobic and anaerobic blood culture bottles and incubated in a BD BACTEC™ automated blood culture system (BD, Franklin Lakes, New Jersey, USA). Growth was detected from both vials, with a time to positivity of 17 hours for the aerobic and 27 hours for the anaerobic culture. Small alpha-hemolytic colonies were observed on blood agar after incubation in both aerobic with 5% CO_2_ enrichment and anaerobic conditions. Microscopy showed Gram-positive cocci that were arranged in chains, and the catalase test was negative.

Identification upto the species level was obtained, using VITEK® MS (v3.0; bioMérieux, Marcy-l'Étoile, France) matrix-assisted laser desorption ionization time-of-flight (MALDI-TOF), which produced a result with a 99.9% confidence value. In-vitro antibiotic susceptibility, according to the European Committee on Antimicrobial Susceptibility Testing (EUCAST) guidelines, was performed. This was done by preparing a 0.5 McFarland suspension in Mueller Hinton broth, from which several Mueller Hinton agar specimens containing 5% horse blood and 20 mg/l ß-NAD (MHF) were inoculated using the Kirby-Bauer method. The relevant MIC strips and antibiotic discs were then placed onto the MHF media, and the following day, the zones of inhibition were interpreted. In-vitro antibiotic susceptibility testing was performed, and MIC values, as seen in Table 1, were obtained for benzylpenicillin, ampicillin, ceftriaxone, vancomycin, and levofloxacin using Liofilchem® MTS™ gradient MIC strips. Clindamycin susceptibility was tested via disk diffusion using the MASTDISCS® Antibiotic Susceptibility Test (AST) (Mast Group Ltd, Bootle, UK), and inducible resistance was excluded.

**Table 1:** MIC values in mg/L of the tested antibiotics MIC: minimum inhibitory concentration

Antibiotic	MIC value
Benzylpenicillin	0.064 mg/L
Ampicillin	0.064 mg/L
Ceftriaxone	0.064 mg/L
Vancomycin	0.25 mg/L
Levofloxacin	0.25 mg/L

Treatment given

The patient received both medical and surgical management to treat infective endocarditis. Since the patient was not acutely ill on presentation, blood cultures were taken, and empiric therapy was not instituted. However, two sets of blood cultures taken on the day of presentation turned positive for *Streptococcus sinensis* within 48 hours. Therefore, targeted anti-microbial therapy could be administered. The choice of antibiotic in this patient was intravenous ceftriaxone 2 g daily.

Ceftriaxone is a β-lactam antibiotic that displays bactericidal activity against both gram-positive and gram-negative aerobic bacteria. The bacterium cultured was a sensitive gram-positive organism, making Ceftriaxone an appropriate choice. In most cases of uncomplicated native valve endocarditis, a four-to-six-week course of intravenous antibiotics is advised.

However, in this case, given that the patient had large vegetations visualized on echocardiography and progressive heart failure, surgical intervention to replace the damaged valve was necessary, even if a full course of antibiotic therapy had not yet been completed. Surgical intervention confirmed that despite both valves being infected, there was no evidence of extra-valvular extension of the infection.

Other indications for surgery in patients with infective endocarditis include a presentation with valve dysfunction causing heart failure, the presence of annular or aortic abscesses, persistent fever, or bacteremia despite seven days of appropriate antibiotics and infected prosthetic valves.

## Discussion

*Streptococcus sinensis* is a viridans group *Streptococcus*, which was first isolated in 2002 from a 42-year-old woman from Hong Kong who was diagnosed with infective endocarditis. Since then, a further two cases have been reported in Hong Kong in 2004, followed by sporadic cases reported in Europe. The case presented here is not only the first case of *S. sinensis* endocarditis in the Maltese Islands but also one of the few cases reported in Europe. To date, cases have only been reported in four other European nations, namely, Italy, France, the United Kingdom and the Netherlands [4-7].

In most cases reported, patients had a history of travel to Hong Kong or a history of rheumatic heart disease. In this case, we could not identify an epidemiological link to Hong Kong, the patient gave no history of rheumatic fever, and no previous echocardiograms were available. Like other viridans *Streptococci*, *S. sinensis*, is likely to be a normal commensal in the human oral cavity [8]. This hypothesis was tested using a molecular surveillance study on saliva from healthy subjects in Hong Kong, which confirmed that *S. sinensis* was detected in 22% of subjects [9]. Our patient reported developing symptoms approximately one month after a dental scaling procedure; it is highly likely that his oral cavity was colonized with *S. sinensis* and transient bacteremia from the procedure resulted in endocarditis.

The prevalence of infective endocarditis in streptococcal bacteremia is group and species-dependent. Despite being *Streptococci* of relatively low virulence, viridans *Streptococci* are the most common cause of infective endocarditis. Their frequent colonization of the oral cavity [10] is attributed to the ability of the bacterium to utilize glucose to synthesize dextrans, which are one of the principal components of dental plaques. Once these plaques are disturbed, commonly in dental procedures, there is a risk of inducing transient and initially asymptomatic bacteremia, followed by the adherence of bacteria to damaged heart valves.

In all cases documented to date, infections with *S. sinensis* have been shown to cause infective endocarditis and have not been identified as a causative agent of any other infection. This would suggest that this bacterium has a predilection for heart valves, particularly those damaged by previous rheumatic heart disease.

Due to highly similar nucleic acid composition, *S. sinensis* could be mistaken for *S. mitis* or *S. sanguinosus*, two closely related viridans *Streptococci*. However, with the introduction of MALDI-TOF, the identification of different species of *Streptococci* is possible, with a 99.8% sensitivity. Identification of viridans *Streptococci *at the species level may be difficult using 16s RNA sequencing because it may fail to detect minor changes in genetic composition. The relatively recent addition of MALDI-TOF to the microbiologists’ arsenal could suggest that other cases of *S. sinensis* may have been misidentified in the past.

In conclusion, this case emphasizes the role of *S. sinensis* as an emerging pathogen involved in infective endocarditis, with no obvious epidemiological link to Hong Kong or other Asian countries. It also highlights the mortality associated with this infection, in keeping with other case reports.

## Conclusions

The main conclusions that can be drawn from this case report are that serious, deep-seated infections do not necessarily present with fulminant signs and symptoms. Second, a high degree of suspicion, primed by a detailed history and thorough examination is necessary for the timely diagnosis of infective endocarditis. Further, this case highlights the importance of precise microbiological diagnosis, which is often useful both for delivering targeted therapy and for prognostication. Finally, the timing of surgical intervention in infective endocarditis, whether emergent, urgent, or elective remains a difficult yet important decision, best taken by a multispecialty team, including a cardiologist, cardiothoracic surgeon, and infectious disease specialist.

## References

[1] d'Almeida S, Reischmann K, Andreß S (2024). Evaluating the Duke Criteria for infectious endocarditis in a single-center retrospective study. Sci Rep.

[2] Murdoch DR, Corey GR, Hoen B (2009). Clinical presentation, etiology, and outcome of infective endocarditis in the 21st century: the International Collaboration on Endocarditis-Prospective Cohort Study. Arch Intern Med.

[3] Zhang Y, Wang J, Zhan Y, Tang R, Wang H, Qin T, Lu Z (2022). Case report: infective endocarditis caused by Streptococcus sinensis: the first case in mainland China and literature review. Front Cardiovasc Med.

[4] Uçkay I, Rohner P, Bolivar I (2007). Streptococcus sinensis endocarditis outside Hong Kong. Emerg Infect Dis.

[5] Faibis F, Mihaila L, Perna S, Lefort JF, Demachy MC, Le Flèche-Matéos A, Bouvet A (2008). Streptococcus sinensis: an emerging agent of infective endocarditis. J Med Microbiol.

[6] Tomlinson JS, Khan S, Curtis S, James R (2020). Streptococcus sinensis causing infective endocarditis in the Netherlands: our experiences from the UK. Eur Heart J Case Rep.

[7] van Ommen AM, Slavenburg S, Diepersloot R, de Vries Feyens CA (2020). Fatal outcome of first case of Streptococcus sinensis in infective endocarditis in the Netherlands: a case report. Eur Heart J Case Rep.

[8] Woo PC, Teng JL, Leung KW, Lau SK, Tse H, Wong BH, Yuen KY (2004). Streptococcus sinensis may react with Lancefield group F antiserum. J Med Microbiol.

[9] Woo PC, Teng JL, Tsang SN, Tse CW, Lau SK, Yuen KY (2008). The oral cavity as a natural reservoir for Streptococcus sinensis. Clin Microbiol Infect.

[10] Bloch S, Hager-Mair FF, Andrukhov O, Schäffer C (2024). Oral streptococci: modulators of health and disease. Front Cell Infect Microbiol.

